# Elevated levels of endothelial-derived microparticles, and serum CXCL9 and SCGF-β are associated with unstable asymptomatic carotid plaques.

**DOI:** 10.1038/srep16658

**Published:** 2015-11-13

**Authors:** Andrew Schiro, Fiona L. Wilkinson, Ria Weston, J. Vincent Smyth, Ferdinand Serracino-Inglott, M. Yvonne Alexander

**Affiliations:** 1 Regional Vascular and Endovascular Unit, Manchester Royal Infirmary, Central Manchester University Hospitals NHS Foundation Trust, Manchester Academic Health Science Centre, Oxford Road, Manchester, UK, M13 9WL; 2 Institute of Cardiovascular Science, Manchester Academic Health Science Centre, University of Manchester, Core Technology Facility, 46 Grafton Street, Manchester, UK, M13 9NT; 3 Translational Science, Healthcare Science Research Institute, Faculty of Science and Engineering, Manchester Metropolitan University, John Dalton Building, Chester Street, Manchester, UK, M1 5GD

## Abstract

Endothelial microparticles (EMPs) are released from dysfunctional endothelial cells. We hypothesised that patients with unstable carotid plaque have higher levels of circulating microparticles compared to patients with stable plaques, and may correlate with serum markers of plaque instability and inflammation. Circulating EMPs, platelet MPs (PMPs) and inflammatory markers were measured in healthy controls and patients undergoing carotid endarterectomy. EMP/PMPs were quantified using flow cytometry. Bioplex assays profiled systemic inflammatory and bone-related proteins. Immunohistological analysis detailed the contribution of differentially-regulated systemic markers to plaque pathology. Alizarin red staining showed calcification. EMPs and PMPs were significantly higher in patients with carotid stenosis (≥70%) compared to controls, with no differences between asymptomatic *vs* symptomatic patients. Asymptomatic patients with unstable plaques exhibited higher levels of EMPs, CXCL9 and SCGF-β compared to those with stable plaques. CXCL9, and SCGF-β were detected within all plaques, suggesting a contribution to both localised and systemic inflammation. Osteopontin and osteoprotegerin were significantly elevated in the symptomatic *vs* asymptomatic group, while osteocalcin was higher in asymptomatic patients with stable plaque. All plaques exhibited calcification, which was significantly greater in asymptomatic patients. This may impact on plaque stability. These data could be important in identifying patients at most benefit from intervention.

Every year around 145,000 carotid endarterectomies (CEA) and 20,000 carotid stenting procedures are performed in Europe^1^ and the US^2^,^3^ Of these, around 12,000 procedures are performed in asymptomatic patients in an attempt to reduce these patients’ risk of a possible stroke. The two landmark randomised trials, Asymptomatic Carotid Atherosclerosis Study (ACAS)^4^ and the Asymptomatic Carotid Atherosclerosis Trial (ACST)^5^ demonstrated that CEA conferred a 50% relative risk reduction in a 5-year risk of stroke in patients with carotid stenosis of ≥70% from 12% to 6%. ACST, the larger study showed that immediate CEA conferred a 4.6% absolute risk reduction in stroke compared to best medical treatment. This equates to 46 strokes prevented per 1,000 operations over a 10 year period. Not all patients with asymptomatic disease go on to develop a stroke, which is thought to be due to the composition of carotid plaques, as outlined in the Oxford plaque study for symptomatic plaques^6^. This study showed a correlation between presence of symptoms, timing of surgery and the morphological characteristics of plaques. Patients who had a CEA shortly after the onset of symptoms had plaques which showed features of instability that included a higher prevalence of fibrous cap rupture, a large lipid core and dense macrophage infiltration^6^.

The natural progression of atherosclerotic disease is characterised by a chronic inflammatory response in the arterial wall. It is well established that elevated systemic proteins and increased expression of inflammatory cytokines are associated with vulnerable plaque^7^,^8^,^9^. However, due to the complex nature of the underlying inflammatory status of these patients, it is unlikely that identifying single molecules or pathways will yield new therapeutic targets to reduce or alleviate progression of atherosclerotic plaque development. Further investigation is needed to understand the direct and specific effects of the inflammatory cytokines and their interaction with other proteins before they become applied as novel biomarkers for use in the clinic. Inflammatory cytokines, which have been shown to be associated with unstable atherosclerotic plaques in previous studies, may help to predict plaque behaviour.

Recently, there has been interest in the presence and role of circulating microparticles as biomarkers of disease^10^,^11^. Microparticles are anucleoid submicron sized fragments (50 nm-1 μm) derived from damaged cell membranes that harbour lipids, microRNAs, and specific proteins that represent the parent cells they originate from^12^, thus acting as carriers of biological information. Endothelial microparticles (EMPs) are complex vesicular structures released from activated or apoptotic endothelial cells^10^,^13^. We postulated that EMPs may be potential biomarkers of patients who have unstable plaques. These patients are thought to be at higher risk of stroke. The aim of this study was to correlate plaque morphology with circulating EMPs and inflammatory cytokines, in order to generate a panel of biomarkers to identify those patients most at risk of plaque rupture.

## Results

### Plaques from symptomatic patients exhibit increased ulceration and haemorrhage

Asymptomatic patients (n = 19), symptomatic patients (n = 51) and healthy age-matched controls (n = 20) with no history of cardiovascular disease, were recruited into the study. Patients with carotid artery disease, from both groups, were matched evenly for age and various risk factors including hypertension, hypercholesterolaemia and diabetes, with a high proportion of both groups receiving statins, anti-platelet and anti-hypertensive drugs (Supplementary Table S1). Of the symptomatic patients, 13 patients had an acute stroke, 31 patients had a TIA, while 7 patients had retinal embolic disease on the ipsilateral side of their carotid stenosis.

Following surgical intervention on the depiction of stenosis, the plaques were graded histologically into two groups; stable and unstable (see methods). In patients with symptomatic carotid artery stenosis, 42 (82.3%) and 9 (17.7%) patients were identified with unstable and stable plaques respectively, whilst in the asymptomatic group, 13 (68.3%) and 6 (31.7%) patients exhibited unstable plaques and stable plaques respectively.

Plaque ulceration was significantly greater in the plaques from the symptomatic group (48 plaques ulcerated, 96%) compared to those from asymptomatic patients (12 plaques ulcerated, 63%; *P* < 0.0008). Intra-plaque haemorrhage (IPH) was defined as an area containing red cells that caused disruption of plaque architecture^14^ and was greater in symptomatic plaques (42 plaques, 82%) compared with asymptomatic plaques (5 plaques, 26%; *P* = 0.01).

### Endothelial microparticle levels correlate with instability in asymptomatic patient plasma

**Figure 1:**
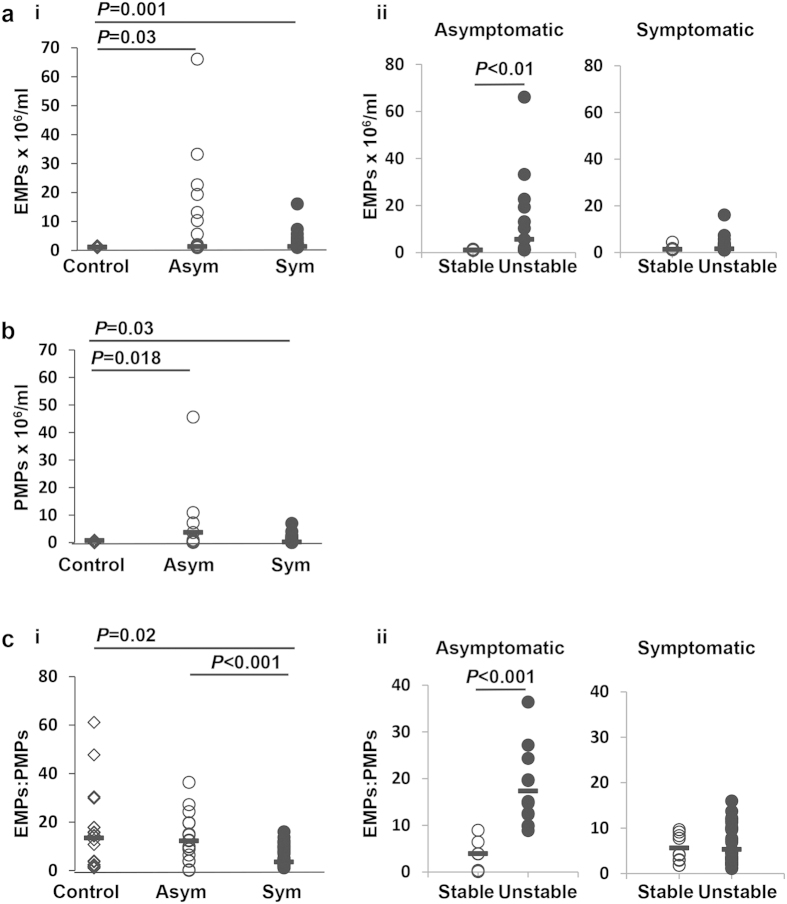
EMPs and EMP:PMP ratio are significantly elevated in asymptomatic patients with unstable plaques. (**a**i and ii) EMPs were defined as CD31+/AnnexinV+/CD42− and counted. (**b**) PMPs were defined as CD31+/AnnexinV+/CD42+ and counted and were significantly elevated in the asymptomatic and symptomatic patient groups compared to healthy controls. (**c**i and ii) The ratio of EMPs to PMPs (CD31+/AnnexinV+/CD42+) was also determined. Bars represent the median. Asym-asymptomatic; Sym-symptomatic; EMP-endothelial-derived microparticles; PMP-platelet-derived microparticles.

Annexin V^+^/CD31^+^/CD42b^−^-EMPs were detected in platelet-poor plasma samples from asymptomatic and symptomatic patients and healthy controls, using flow cytometry (Supplementary Fig. S1). EMPs were significantly elevated in both asymptomatic and symptomatic groups, compared to healthy controls (*P* = 0.03 and *P* = 0.001, respectively) but no differences were detected between the asymptomatic and symptomatic groups (Fig. 1ai).

Each patient group was subdivided based on plaque stability. Significantly elevated EMPs were detected in asymptomatic patients with unstable plaques compared to those with stable plaques (*P* < 0.01), while no such distinction was apparent in symptomatic patients (Fig. 1aii). Furthermore, EMP levels were significantly higher in patients with unstable plaques compared to those with stable plaques, when both the asymptomatic and symptomatic patient groups were combined (*P* = 0.035; Supplementary Fig. S3), as well as showing a weak correlation (r = 0.424; *P* < 0.001) between plaque grade and EMPs.

### A higher EMP to PMP ratio is associated with plaque instability in asymptomatic patients

Annexin V^+^/CD31^+^/CD42b^+^-platelet microparticles (PMPs) were significantly higher in the asymptomatic and symptomatic patient groups compared to healthy controls (*P* = 0.018 and *P* = 0.03), respectively (Fig. 1b). These PMP data strongly correlated with increasing EMPs (r = 0.981; *P* < 0.001), however no differences were detected between the asymptomatic and symptomatic groups. In addition, there was no distinction in PMP levels between patients with stable and unstable plaques in either patient group nor when asymptomatic and symptomatic groups were combined. When the ratio of EMPs to PMPs (EMP:PMP) was measured, it was found that the ratio was significantly lower in the symptomatic group compared to the asymptomatic group and healthy controls (*P* = <0.001 and *P* = 0.02 respectively; Fig. 1ci). In the asymptomatic patient group, the EMP:PMP ratio was significantly higher in patients with unstable plaques compared to the those with stable plaques (Fig. 1cii), but again no distinction of this nature was detected in the symptomatic group.

### Systemic and carotid artery plaque inflammatory profiling

**Figure 2:**
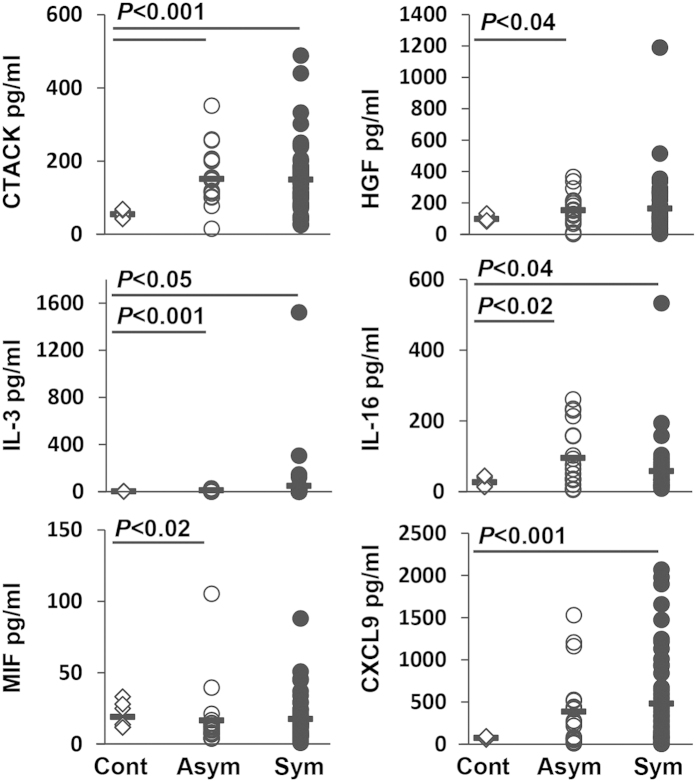
CTACK, IL-3 and IL-16 were significantly elevated in both asymptomatic and symptomatic groups compared to healthy controls. Data were analysed using Kruskal Wallis statistical test. Bars represent the median. Cont; healthy control (n = 20), Asym; asymptomatic (n = 19), Sym; symptomatic (n = 51).

We next examined the relationship between plaque vulnerability and inflammatory biomarkers using pre-operative blood and tissue specimens. From the panel of 21 systemic markers of inflammation analysed, six were differentially regulated in the asymptomatic and symptomatic groups compared to controls. CTACK, IL-3, IL-16 and HGF were significantly higher in asymptomatic patient serum compared to healthy controls (*P* < 0.02), with a trend towards elevation of CXCL9 and a significant reduction in MIF (Fig. 2). CTACK, IL-3, IL-16 and CXCL9 were significantly elevated in symptomatic patient serum compared to healthy controls, with a trend towards an increase in HGF (Fig. 2). No significant differences were detected between asymptomatic and symptomatic patients or in the levels of IL-2Rα, SCGF-β, IL-18, SDF-1α or SCF between any groups. IL-1α, GRO-α, IFN-α2, LIF, MCP-3, M-CSF, β-NGF, TNF-β and TRAIL were below the level of detection.

**Figure 3:**
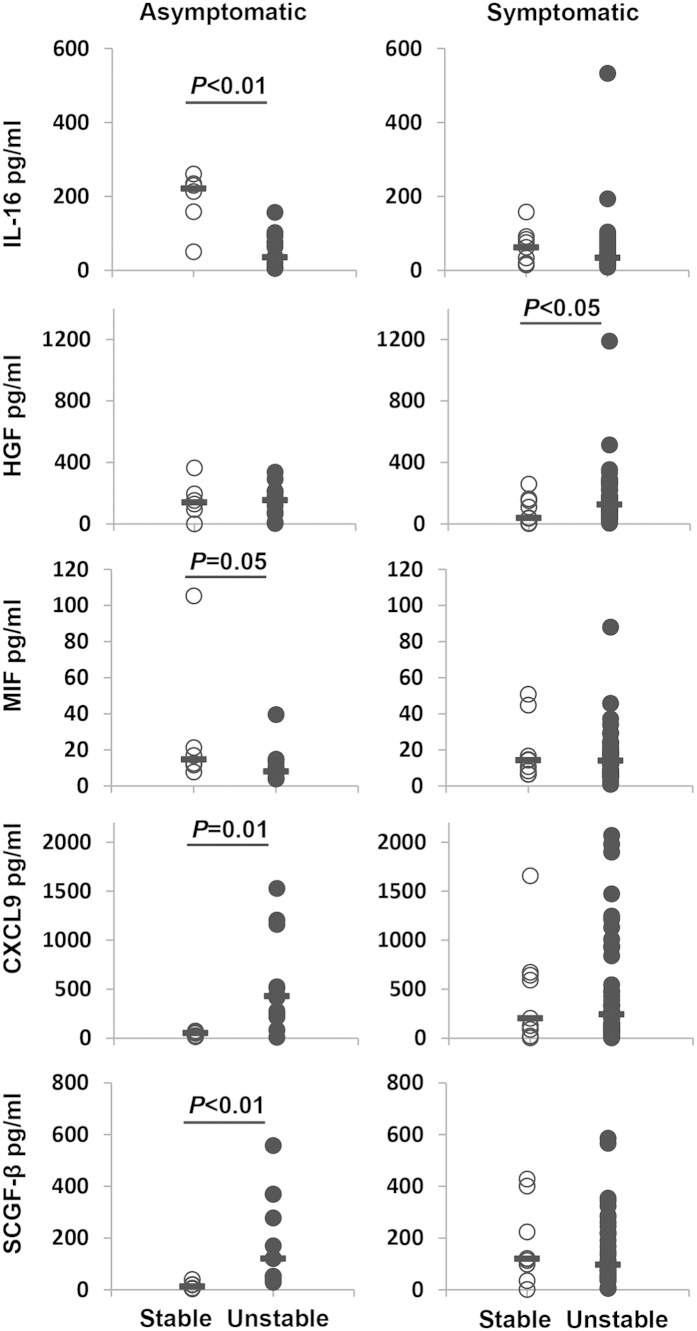
Bioplex analysis of patient serum. CXCL9 and SCGF-β are elevated in asymptomatic patients with unstable plaques, whereas IL-16 and MIF were reduced. HGF was increased in symptomatic patients with unstable plaque. Data were analysed using Mann-Whitney U statistical test. Bars represent the median.

Furthermore, inflammatory markers were compared between unstable and stable plaques in each patient group. In asymptomatic patients, CXCL9 and SCGF-β were significantly elevated (*P* = <0.01) in patients with unstable compared to stable plaques, whereas IL-16 and MIF were significantly elevated in the stable plaque group (Fig. 3). In addition, EMPs exhibited a strong negative correlation with the level of SCGF-β (r = −0.943; *P* < 0.005) and IL-16 (r = −0.829; *P* < 0.05) in asymptomatic patients with stable plaques. HGF was significantly elevated in symptomatic patients with unstable plaque (*P* < 0.05) with no other differences detected within the symptomatic group (Fig. 3). IL-2Rα, IL-12P40, CTACK, IL-3, IL-18, or SCF-1 showed no difference in patients with stable and unstable plaques in either the asymptomatic or symptomatic group. When we compared all patients with stable versus unstable plaques, CXCL9 (*P* = 0.040) was significantly elevated and IL-16 (*P* = 0.014) was significantly lower in patients with unstable plaques.

**Figure 4:**
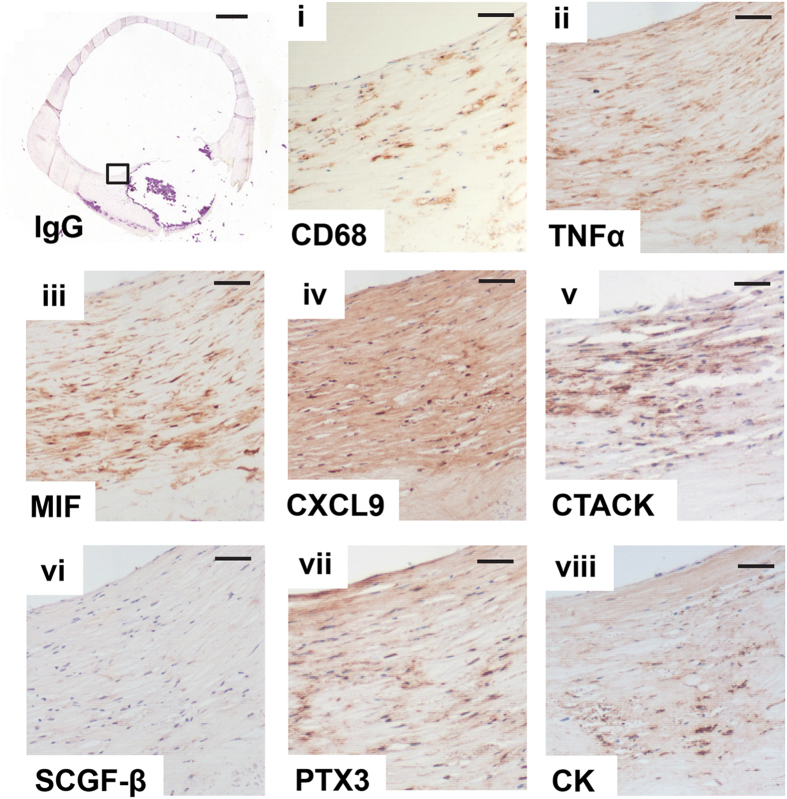
Inflammatory markers are expressed within carotid endarterectomy plaques. Square box in the negative IgG control is enlarged in the serial sections presented in micrographs (**i–viii**). Consecutive sections from a representative patient sample stained positive for inflammatory markers (**i**) CD68 and (**ii**) TNFα-tumour necrosis factor-alpha. The expression of a panel of cytokines, (**iii**) MIF-macrophage migration inhibitory factor, (**iv**) CXCL9, (**v**) CTACK-cutaneous T-cell-attracting chemokine, (**vi**) SCGF-β-stem cell growth factor beta, (**vii**) PTX3-pentraxin-related protein and (**viii**) cathepsin K was also detected within the plaque. IgG bar = 1000 μm; (**i–viii**) bars  = 50 μm.

The plaque composition, cellularity, severity of inflammation, and atheroma-associated macrophages and foam cells were analysed. CD68-positive macrophages were detected in all of the CAE specimens analysed, particularly within the shoulders of the plaque (Fig. 4), and of note, the inflammatory nature of these macrophages could be inferred by the presence of TNF-α immunoreactivity in the same vicinity in consecutive sections. To determine whether the up-regulated systemic markers of inflammation present in blood samples of these patients might also influence localised plaque pathology, we assessed their presence within the plaques. MIF, CXCL9, CTACK and SCGF-β were detected in lesions from both asymptomatic and symptomatic groups with no differences between stable and unstable plaques, nor between asymptomatic and symptomatic groups. All IgG controls were negative. Given the close association between thin plaque atheroma^15^, macrophage activity and cathepsin K (CatK) expression, we compared the carotid atheroma specimens for CatK staining and demonstrated expression within the fibrous cap of all specimens with little, if any distinction between groups. In addition, given the recent interest in pentraxin (PTX3) as a new biomarker for inflammatory vascular disease, our data support the findings of others^16^ where we demonstrate, not only a positive correlation between the expression of PTX3 in the plaque and the presence of macrophage cells, but also with a range of inflammatory mediators (Fig. 4).

### Plaques from asymptomatic patients exhibit extensive calcification

**Figure 5:**
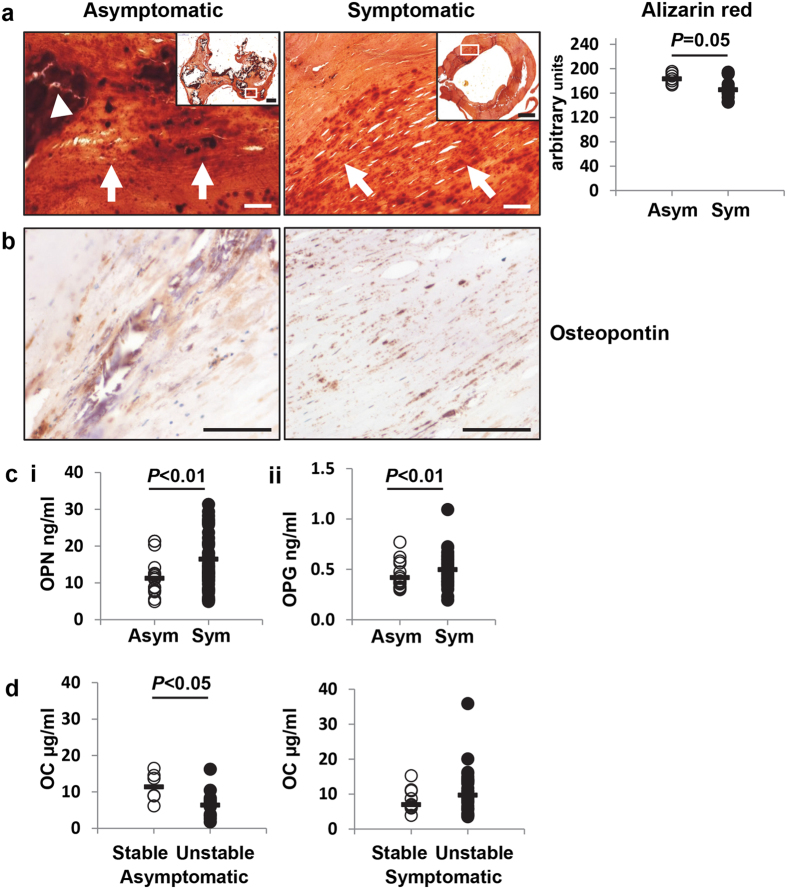
Plaques from asymptomatic patients were more calcified than from symptomatic patients. (**a**) Tissue sections were stained with Alizarin red to highlight areas of calcification, which appeared as spicules (arrows) or as large blocks (arrow head). Plaques from asymptomatic patients (Asym; n = 8) exhibited significantly more Alizarin red staining compared to plaques from symptomatic patients (Sym; n = 11). (**b**) Osteopontin (OPN) was detected in similar areas of consecutive sections. (c and d) Bone-related markers were differentially regulated in patient serum. Data were analysed using Mann-Whitney U statistical test. Bars represent the median. Inset image; bar = 1000 μm. Main image; bar = 100 μm. Asym-asymptomatic; Sym-symptomatic.

Plaques from asymptomatic and symptomatic patients exhibited substantial amounts of vascular calcification, evident both as small spicules and as large blocks, highlighted by the Alizarin red staining (Fig. 5a). Quantification of the Alizarin red staining revealed that the plaques from asymptomatic patients had significantly more positive mineralisation than the symptomatic group (*P* = 0.05; Fig. 5a). Osteopontin (OPN), a bone related protein, was also detected in close proximity to the calcified areas (Fig. 5b), thus confirming our previous observations, of the association between OPN positive staining within the vicinity of calcification in the vessel wall^17^,^18^.

Therefore, we questioned whether proteins involved in bone metabolism may be differentially regulated in the circulation of these patients. OPN (Fig. 5ci) and OPG (Fig. 5cii), inhibitors of bone formation, were both elevated in the serum of symptomatic patients compared to asymptomatic patients (*P* < 0.01), with no difference detected between stable and unstable plaques in either group. Osteocalcin (OC), involved in bone formation, was found to be elevated in stable plaques in the asymptomatic group (*P* < 0.05) but not in the symptomatic group (Fig. 5d). No differences were found in the levels of IL-6, TNF-α, or sclerostin.

## Discussion

Current clinical practice relies on the patient history and medical imaging in the form of Duplex ultrasound, computed tomography angiography and magnetic resonance angiography to select patients with carotid disease that may benefit from surgery. The role of inflammatory markers associated with cardiovascular disease has yet to gain a role in the selection process, when trying to decide which patients will benefit from intervention. It is likely, that in the future, serum biomarkers may become an important clinical tool that could revolutionise the treatment of patients with cardiovascular disease such as those patients with asymptomatic carotid stenosis.

In this study no distinction could be made on actual EMP numbers between symptomatic and asymptomatic patient groups, in agreement with Leroyer *et al.*^19^ nor on PMP levels. In the unit from where we obtained our carotid plaques, surgery is not routinely offered to all patients with asymptomatic disease with ≥70% stenosis. Surgery is only performed to the subgroup of patients who we feel are at high risk of developing a stroke compared to the standard asymptomatic group of patients, for example, patients with ultrasound features of unstable plaques, patients who have a history of a transient ischaemic attack (>6 months) or patients requiring coronary artery bypass graft surgery. 68% of patients in our asymptomatic group had unstable plaques compared to 82% in the symptomatic group. It is therefore not surprising that we found no differences in EMP and PMP levels between the two groups. In view of this finding, the remainder of our analysis largely focused on comparing stable and unstable plaque groups.

Reports show that EMPs are elevated in response to endothelial damage^7^,^20^ as well as other cardiovascular-related diseases^21^,^22^. When we subdivided patients based on the nature of their plaques being stable or unstable, we detected a significant elevation of EMPs in the unstable plaques. When this analysis was repeated within the asymptomatic group, a difference in EMPs between stable and unstable plaques was still present and significant. This is in keeping with the findings of Wekesa *et al.*^23^, and Sarlon-Bartoli *et al.*^24^. It is interesting to note that EMPs have been shown to be elevated in patients with familial hypercholesterolaemia (FH) compared to non-FH patients with hypercholesterolaemia^25^, suggesting that high levels of cholesterol in the long-term may induce EMP release.

We found no changes in PMP levels in relation to stable or unstable plaques in either patient group, consistent with Wekesa *et al.*^23^ Interestingly, the ratio of EMPs to PMPs was lower in the symptomatic patients compared to asymptomatic patients and controls. However, further analysis showed that the EMP:PMP ratio was significantly higher in asymptomatic patients with unstable plaques. This finding may reflect a more inflammatory environment with the potential for plaque instability and warrants further functional investigation to identify a mechanism. Other groups have shown that leukocyte-derived MPs were elevated in patients with unstable plaques^24^ and lipid-rich plaques in patients with FH and hypercholesterolaemia^25^. These data highlight the important contribution of MPs derived from other cell types.

These data could have significant impact, since EMPs are not thought to give rise to symptoms *per se,* but instead act as a silent indicator of internal damage to the integrity of the endothelium, thus adding strength to the concept of EMPs being a biomarker of plaque instability in the asymptomatic group. There is a growing need for the use of biomarker discovery in plasma as a means of developing a non-invasive diagnostic/prognostic test for stroke susceptibility. We hypothesized that patients at high risk of future events could be identified prospectively by a combination of high risk plaque features, levels of microparticles and a set of circulating blood biomarkers.

Next, we investigated levels of different serum cytokines to establish the inflammatory profile of the patients. Using a cytokine bead array-approach, we identified several novel biomarkers, which were differentially up and down-regulated in the different patient groups. CTACK, IL-3 and IL-16, which serve as chemo-attractants for different immune cells that are involved in plaque development, were significantly elevated in both symptomatic and asymptomatic patient serum compared to healthy controls. In contrast, MIF, was shown to be just significantly down-regulated in the asymptomatic group *vs* controls, which may be due to patient variability and should be investigated further in larger studies. However, this could prove to be an interesting finding, given that MIF shows properties associated with clot retraction^26^. The finding that HGF was raised in the symptomatic group supports our previous studies on elevated expression of HGF in carotid disease patients^27^, and other reports where hepatocyte growth factor (HGF) has been shown to have a pathophysiological role in disease progression^27^. Serum CXCL9 levels have also been associated independently with coronary artery calcification score after adjusting for traditional cardiovascular risk factors, suggesting that CXCL9 may be used in a panel as a novel biomarker of atherosclerotic plaque burden in humans^28^.

Since the presence of activated macrophages and inflammation is a feature of rupture-prone plaques, and the fact that current imaging techniques cannot be reliably used for the detection of a vulnerable plaque in the clinic, a combination approach using imaging and serum biomarkers for detecting high-risk plaques and preventing acute cardiovascular events, could have clinical benefit for the future development of a point-of-care prognostic device. Therefore, we interrogated the patient groups to determine whether we could correlate serum biomarkers with plaque vulnerability. We now demonstrate that, CXCL9 and SCGF-β were significantly elevated in serum from patients with unstable plaques compared to those with stable plaques, in the asymptomatic group, potentially increasing the invasion of inflammatory cells into the plaque, thus contributing to the instability. When all stable and unstable plaques from both groups were analysed with regards to inflammatory markers, CXCL9 was the only cytokine significantly elevated. Furthermore, there was a strong negative correlation between the level of SCGF-β and EMPs in asymptomatic patients with stable plaque, supporting the concept of an elevated inflammatory/EMP profile, for the unstable group of patients at most risk of stroke. In addition, IL-16 was down-regulated in serum of patients with unstable *vs* stable plaques, the reason for this is unclear, however, the Interleukins are known to be regulatory proteins which can either accelerate or inhibit inflammatory processes, thus acting as damaging, protective or neutral agents, most likely determined by their concentration and other regulatory factors being modulated in the disease process^29^.

The transition from a stable to an unstable plaque puts the patients at risk of thromboembolism and stroke. It is characterised by increased neovascularization and foam cell infiltration, as shown in our previous study^27^. Although intensive medical therapy and carotid endarterectomy have been shown to reduce stroke and death rates in asymptomatic stenosis, it is recognised that patients with low to intermediate artery stenosis can still succumb to ischemic events, providing the impetus to consider plaque composition as an important feature of atherosclerosis. The findings from studies of this nature could in the future contribute to the selection of patients for CEA surgery. This fits with the current strategy for precision medicine.

We investigated the presence of the same biomarkers in plaques to establish if we would find a correlation between systemic and local expression with EMPs and plaque morphology to enable an identification of patients at high risk of future events. The inflammatory markers were present in both types of plaques as expected, adding further strength to the value of systemic markers for the distinction of stable and unstable plaques. In addition, we investigated the collagenase, cathepsin K, since it has been described as an attractive target for plaque regression and selective inhibitors are already being used in phase III clinical trials^30^. It is not surprising to find the presence of this collagenase in all plaques, since hemodynamics play such a key role in atherosclerotic development and the carotid endarterectomy specimens are taken from regions of low and oscillatory shear stress, where endothelial cells have been shown to contribute to proteolytic vascular remodelling by upregulating cathepsins^31^. These findings are in accordance with the results previously seen by Lutgens *et al.*, who demonstrated cathepsin K expression is up-regulated in advanced lesions up to 28 fold^32^. In addition, it is interesting to note that both Cathepsin-K and macrophage migration inhibitory factor (MIF) have been shown to be involved in bone metabolism^33^ and we find the presence of both molecules in association with mineral deposition in plaques with evidence of calcification. PTX3 expression was also detected in the lesions, alongside other mediators of inflammation.

Elevated calcification was apparent in the plaques from the asymptomatic group compared to the symptomatic group, whether this affords increased stability remains debatable. In light of these findings, we investigated circulating bone markers and found significantly raised levels of OPN and OPG in the symptomatic group *vs* asymptomatic patients, which adds strength to our histological findings, as both OPN and OPG are inhibitors of mineralisation, supporting the reduced mineralisation detected in the symptomatic patients. Furthermore OC, involved in bone formation, was elevated in asymptomatic patients with stable plaques, lending credence to the idea that calcification could have a stabilising influence. Although tissue analysis of these pro-inflammatory cytokines will not be used clinically, identifying specific cellular and signalling pathways in plaque development will provide a deeper insight into mechanisms of the disease process and could provide a target to be used with non-invasive imaging to enhance the diagnostic and predictive opportunities in this patient group. Although the data set in this study are small, it highlights the challenges associated with patient-to-patient variability and the need to collect not only serological, cellular and molecular analyses of clinical samples, but to include genetic information and utilise large scientific data sets, obtained from state-of-the-art omic technologies.

**Figure 6:**
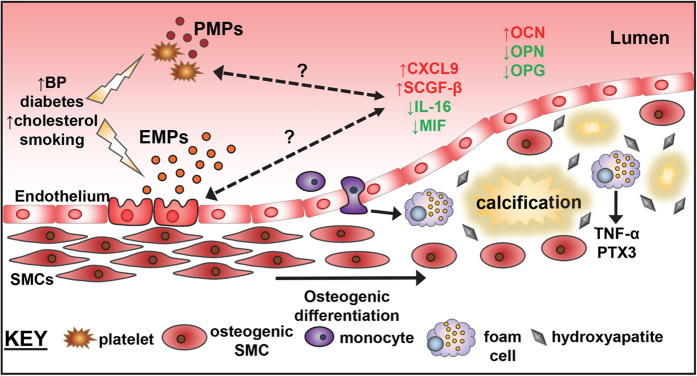
A schematic representation of the key factors involved in plaque instability in asymptomatic patients. Endothelial cells and platelets are activated by various cardiovascular disease risk factors and release microparticles into the circulation. During plaque development, smooth muscle cells (SMCs) can differentiate from a contractile to a secretory phenotype, depositing a calcified matrix. Both processes may be exacerbated by the cytokines modulated during disease pathology. OC-osteocalcin; OPN-osteopontin; OPG-osteoprotegerin; TNF-tumour necrosis factor-alpha; PTX3-pentraxin-related protein; SCGF-β-stem cell growth factor; SMC-smooth muscle cell; EMP-endothelial microparticle; PMP-platelet microparticle.

A biomarker-based risk stratification for carotid disease may greatly assist clinicians to determine which patients are more likely to have a cerebrovascular event. In turn, this reduces the number of prophylactic carotid endarterectomies performed and their associated complications. Our data, summarised in Fig. 6, may contribute to the future development of a risk-profiling tool for assessment of EMPs and a specific inflammatory marker signature, which can be used in combination with current imaging to detect plaque vulnerability and stroke susceptibility.

## Methods

### Subjects

Seventy patients, who underwent a carotid endarterectomy, at the Manchester Royal Infirmary Vascular Unit between April 2012 and April 2013, were studied prospectively. Duplex ultrasonography was used to determine the degree of stenosis in the carotid artery prior to surgery (Supplementary Table S2). Indications for surgery included symptomatic carotid artery stenosis of ≥50% stenosis or asymptomatic stenosis of ≥70%. All participants gave informed consent to the study, which was approved by the Yorkshire & The Humber - Leeds West NRES Committee (REC reference 12/YH/0107), and the protocol was carried out in accordance with their approved guidelines. Patient demographics are summarised in Supplementary Table S1. Patients who had clinical evidence of infection, or had received cancer treatment in the last 6 months were excluded. Symptomatic patients (n =  51) were those who experienced an acute stroke, transient ischaemic attack (TIA), or retinal embolic disease within one month from surgery, whilst asymptomatic patients (n = 19) exhibited no neurological symptoms in the 6 months preceding surgery. A group of 20 healthy age-matched subjects who showed no cardiovascular risk factors and were not on any medication were recruited as controls for blood analysis.

### Blood sample preparation

Peripheral venous fasting blood samples, were collected pre-operatively on the day of surgery using a 21G needle (BD Vacutainer ® systems). Bloods samples were centrifuged within 1 hour of blood letting. Platelet-poor plasma (PPP) was isolated by centrifugation of citrate tubes at 1700 × g for 10 minutes at 4 °C. The plasma layer was further centrifuged at 20,000 × g for 10 minutes at 4 °C and after discarding the platelet pellet, the PPP was stored at −80 °C for microparticle analysis. Blood was also collected in a serum tube (BD) and left to clot for 30 minutes at room temperature. Serum was obtained by centrifugation at 4 °C at 1700 × g for 15 minutes and the supernatant was stored at −80 °C for inflammatory marker profiling.

### Microparticle analysis by flow cytometry

To quantify the levels of EMPs and PMPs within samples, PPP samples were mixed with 10 μm flow counting beads (Beckman Coulter, UK) in the presence of calcium-rich buffer (eBioscience, UK). Analysis of the MP populations was carried out using a cocktail of phycoerythrin (PE) -conjugated anti-human CD31 (BD Bioscience, UK), allophycocyanin (APC) -conjugated anti-human CD42b (BD Bioscience, UK) and efluor450 annexin-V marker (eBioscience, UK) as previously described^34^. MPs were analysed using a Cyan flow cytometer (BD Biosciences) according to their size and fluorescence using a logFS-logSS plot. EMPs were characterised and enumerated as Annexin V+/CD31+/CD42b- events, and PMPs as Annexin V+/CD31+/CD42b+ events (Supplementary Fig. S1).

### Cytokine and bone metabolism marker analysis

A panel of 21 cytokines were analysed in patient serum samples using the Bio-plex Pro™ Human Cytokine 21-Plex immunoassay (Bio-Rad Laboratories) based on Luminex xMAP™ technology, according to the manufacturer’s protocol. These included, interleukin-1alpha (IL-1α), interleukin-2 receptor-alpha (IL-2Rα), interleukin-3 (IL-3), interleukin-12p40 (IL-12p40), interleukin-16 (IL-16), interleukin-18 (IL-18), cutaneous T-cell-attracting chemokine (CTACK), growth regulated oncogene-alpha (GROα), hepatocyte growth factor (HGF), interferon alpha-2 (IFN-α2), leukocyte inhibitory factor (LIF), monocyte chemoattractant protein-3 (MCP-3), monocyte colony stimulating factor (M-CSF), macrophage migration inhibitory factor (MIF), monokine induced by gamma interferon (MIG or CXCL9), nerve growth factor-beta (β-NGF), stem cell factor (SCF), stem cell growth factor-beta (SCGF-β), stromal cell-derived factor-1 alpha (SDF-1α) and tumour necrosis factor-beta (TNF- β). Samples were analysed in duplicate and data were collected and analysed using the Bio-plex™ Manager Software (Bio-rad Laboratories) version 6.0. Six bone-related markers were analysed using the Milliplex MAP Human Bone Magnetic Bead Panel (Millipore) which included IL-6, tumour necrosis factor-alpha (TNF-α), osteoprotegerin (OPG), osteocalcin (OC), osteopontin (OPN) and sclerostin. Samples were analysed using the Milliplex Analyser (Millipore).

### Carotid plaque collection and analysis

Fresh carotid plaques were obtained during surgery. Samples were fixed in 4% formaldehyde and paraffin embedded. 7 μm sections were mounted on positively charged slides (Superfrost, Germany). Five haematoxylin and eosin (H&E) stained plaques sections from each specimen were examined microscopically to determine features of plaque stability; fibrous plaque with a thick intact cap and instability; thin cap, large lipid core, intra-plaque haemorrhage and presence of inflammatory cells (CD68-positive macrophages) according to the Oxford plaque study^6^. Plaque sections were stained with anti-CD68 antibody (Dako), anti-TNFα antibody (Santa Cruz), anti-MIF antibody, MIG, CXCL9 and Cathepsin K (Abcam), anti-OPN, anti-MIF, anti-SCGF and CTACK (R&D Systems) and anti-PTX3 (Abnova) as described previously. Intact CD68-positive cells were counted using Image J software (NIH, Bethesda, MD) in the entire plaque section. Absolute count was divided by the plaque area to produce a value of CD68-positive cells per mm^2^ (Supplementary Fig. S2). Alizarin red stained sections were imaged using a Panoramic SCAN with Zeiss Plan-apochromat 20×/0.8 objective, (3D Histotech/Laser2000, Ringstead, UK). Images were taken at x20 magnification using Pannoramic Viewer software (3D Histech) from the whole tissue section and the mean intensity of staining was determined using Image J software for each patient in the asymptomatic and symptomatic groups.

### Statistical analyses

Statistical analyses of data were carried out using SPSS version 19.0 (SPSS, Inc, Chicago, IL) using either Kruskal Wallis, Mann-Whitney U or Spearman’s correlation coefficient non-parametric tests, taking *P* < 0.05 as statistically significant. Chi-squared test was used for categorical data.

## Additional Information

**How to cite this article**: Schiro, A. *et al.* Elevated levels of endothelial-derived microparticles, and serum CXCL9 and SCGF-β are associated with unstable asymptomatic carotid plaques. *Sci. Rep.* **5**, 16658; doi: 10.1038/srep16658 (2015).

## Supplementary Material

Supplementary Information

## References

[b1] Rudarakanchana N. *et al.* Current practice of carotid endarterectomy in the UK. Br J Surg 99, 209–216 (2012). 22190246 10.1002/bjs.7810

[b2] National Institute of Neurological Disorders and Stroke; Questions and Answers About Carotid Endarterectomy. http://www.ninds.nih.gov/disorders/stroke/carotid_endarterectomy_backgrounder.htm, (2012), (Date of access:07/05/2012).

[b3] Choi J. C., Johnston S. C. & Kim A. S. Early outcomes after carotid artery stenting compared with endarterectomy for asymptomatic carotid stenosis. Stroke 46, 120–125 (2015). 25424479 10.1161/STROKEAHA.114.006209

[b4] Endarterectomy for asymptomatic carotid artery stenosis. Executive Committee for the Asymptomatic Carotid Atherosclerosis Study. JAMA 273, 1421–1428 (1995). 7723155

[b5] Halliday A. *et al.* 10-year stroke prevention after successful carotid endarterectomy for asymptomatic stenosis (ACST-1): a multicentre randomised trial. Lancet 376, 1074–1084 (2010). 20870099 10.1016/S0140-6736(10)61197-X PMC2956884

[b6] Redgrave J. N., Lovett J. K., Gallagher P. J. & Rothwell P. M. Histological assessment of 526 symptomatic carotid plaques in relation to the nature and timing of ischemic symptoms: the Oxford plaque study. Circulation 113, 2320–2328 (2006). 16651471 10.1161/CIRCULATIONAHA.105.589044

[b7] Malaud E. *et al.* Local carotid atherosclerotic plaque proteins for the identification of circulating biomarkers in coronary patients. Atherosclerosis 233, 551–558 (2014). 24530963 10.1016/j.atherosclerosis.2013.12.019

[b8] Tomey M. I., Narula J. & Kovacic J. C. Advances in the understanding of plaque composition and treatment options: year in review. J Am Coll Cardiol 63, 1604–1616 (2014). 24583311 10.1016/j.jacc.2014.01.042

[b9] Ait-Oufella H., Taleb S., Mallat Z. & Tedgui A. Recent advances on the role of cytokines in atherosclerosis. Arterioscler Thromb Vasc Biol 31, 969–979 (2011). 21508343 10.1161/ATVBAHA.110.207415

[b10] Schiro A. *et al.* Endothelial microparticles as conveyors of information in atherosclerotic disease. Atherosclerosis 234, 295–302 (2014). 24721189 10.1016/j.atherosclerosis.2014.03.019

[b11] Lacroix R. & Dignat-George F. Microparticles: new protagonists in pericellular and intravascular proteolysis. Semin Thromb Hemost 39, 33–39 (2013). 23303566 10.1055/s-0032-1333310

[b12] Dalli J. *et al.* Heterogeneity in neutrophil microparticles reveals distinct proteome and functional properties. Mol Cell Proteomics 12, 2205–2219 (2013). 23660474 10.1074/mcp.M113.028589 PMC3734580

[b13] Mause S. F. & Weber C. Microparticles: protagonists of a novel communication network for intercellular information exchange. Circ Res 107, 1047–1057 (2010). 21030722 10.1161/CIRCRESAHA.110.226456

[b14] Bassiouny H. S. *et al.* Critical carotid stenoses: morphologic and chemical similarity between symptomatic and asymptomatic plaques. J Vasc Surg 9, 202–212 (1989). 2537432

[b15] Chatzizisis Y. S. *et al.* Augmented expression and activity of extracellular matrix-degrading enzymes in regions of low endothelial shear stress colocalize with coronary atheromata with thin fibrous caps in pigs. Circulation 123, 621–630 (2011). 21282495 10.1161/CIRCULATIONAHA.110.970038 PMC3066078

[b16] Shindo A. *et al.* Inflammatory biomarkers in atherosclerosis: pentraxin 3 can become a novel marker of plaque vulnerability. PLoS One 9, e100045 (2014). 24936646 10.1371/journal.pone.0100045 PMC4061039

[b17] Alexander M. Y. *et al.* Identification and characterization of vascular calcification-associated factor, a novel gene upregulated during vascular calcification *in vitro* and *in vivo*. Arterioscler Thromb Vasc Biol 25, 1851–1857 (2005). 15994437 10.1161/01.ATV.0000175750.94742.46

[b18] Wilkinson F. L. *et al.* Contribution of VCAF-positive cells to neovascularization and calcification in atherosclerotic plaque development. The Journal of pathology 211, 362–369 (2007). 17154367 10.1002/path.2114 PMC1868967

[b19] Leroyer A. S. *et al.* Cellular origins and thrombogenic activity of microparticles isolated from human atherosclerotic plaques. J Am Coll Cardiol 49, 772–777 (2007). 17306706 10.1016/j.jacc.2006.10.053

[b20] Lanzino G., Couture D., Andreoli A., Guterman L. R. & Hopkins L. N. Carotid endarterectomy: can we select surgical candidates at high risk for stroke and low risk for perioperative complications? Neurosurgery 49, 913–923; discussion 923-914 (2001). 11564254 10.1097/00006123-200110000-00025

[b21] Viera A. J., Mooberry M. & Key N. S. Microparticles in cardiovascular disease pathophysiology and outcomes. J Am Soc Hypertens 6, 243–252 (2012). 22789878 10.1016/j.jash.2012.06.003

[b22] Ueba T. *et al.* Plasma level of platelet-derived microparticles is associated with coronary heart disease risk score in healthy men. J Atheroscler Thromb 17, 342–349 (2010). 20379056 10.5551/jat.2964

[b23] Wekesa A. L. *et al.* Predicting carotid artery disease and plaque instability from cell-derived microparticles. EJVES 48, 489–495 (2014). 10.1016/j.ejvs.2014.08.007 25218652

[b24] Sarlon-Bartoli G. *et al.* Plasmatic level of leukocyte-derived microparticles is associated with unstable plaque in asymptomatic patients with high-grade carotid stenosis. J Am Coll Cardiol 62, 1436–1441 (2013). 23707318 10.1016/j.jacc.2013.03.078

[b25] Suades R., Padró T., Alonso R., Mata P. & Badimon L. Lipid-lowering therapy with statins reduces microparticle shedding from endothelium, platelets and inflammatory cells. Thromb Haemost 110, 366–377 (2013). 23740299 10.1160/TH13-03-0238

[b26] Wirtz T. H. *et al.* Platelet-derived MIF: A novel platelet chemokine with distinct recruitment properties. Atherosclerosis 239, 1–10 (2015). 25561410 10.1016/j.atherosclerosis.2014.12.039

[b27] Chowdhury M. *et al.* A comparative study of carotid atherosclerotic plaque microvessel density and angiogenic growth factor expression in symptomatic versus asymptomatic patients. Eur J Vasc Endovasc Surg 39, 388–395 (2010). 20122857 10.1016/j.ejvs.2009.12.012

[b28] Yu H. T. *et al.* Serum monokine induced by gamma interferon as a novel biomarker for coronary artery calcification in humans. Coron Artery Dis, 26, 317–321 (2015). 25756330 10.1097/MCA.0000000000000236

[b29] Fisman E. Z., Motro M. & Tenenbaum A. Cardiovascular diabetology in the core of a novel interleukins classification: the bad, the good and the aloof. Cardiovasc Diabetol 2, 11, (2003). 14525620 10.1186/1475-2840-2-11 PMC212422

[b30] Newby A. C. Proteinases and plaque rupture: unblocking the road to translation. Curr Opin Lipidol 25, 358–366 (2014). 25089553 10.1097/MOL.0000000000000111

[b31] Platt M. O. *et al.* Expression of cathepsin K is regulated by shear stress in cultured endothelial cells and is increased in endothelium in human atherosclerosis. Am J Physiol Heart Circ Physiol 292, H1479–1486 (2007). 17098827 10.1152/ajpheart.00954.2006

[b32] Lutgens E. *et al.* Disruption of the cathepsin K gene reduces atherosclerosis progression and induces plaque fibrosis but accelerates macrophage foam cell formation. Circulation 113, 98–107 (2006). 16365196 10.1161/CIRCULATIONAHA.105.561449

[b33] Mandelin J. *et al.* Human osteoblasts produce cathepsin K. Bone 38, 769–777 (2006). 16337236 10.1016/j.bone.2005.10.017

[b34] Parker B. *et al.* Suppression of inflammation reduces endothelial microparticles in active systemic lupus erythematosus. Ann Rheum Dis 73, 1144–1150 (2013). 23644670 10.1136/annrheumdis-2012-203028 PMC4033120

