# Supplementary material for: Elevated levels of endothelial-derived microparticles, and serum CXCL9 and SCGF-β are associated with unstable asymptomatic carotid plaques
Source: Sci Rep. 2015 Nov 13;5:16658. doi: 10.1038/srep16658 (PMC4643236; doi:10.1038/srep16658)
Supplement: Supplementary Information [file srep16658-s1.pdf]

Elevated levels of endothelial-derived microparticles and serum CXCL9 and SCGF-β are associated with unstable asymptomatic carotid plaques.

+A.Schiro MRCS[1,2], +F.L Wilkinson PhD[3], R Weston PhD[3], J.V. Smyth FRCS[1], F. Serracino-Inglott, FRCS[1,2,3], *M.Y. Alexander PhD[2,3].
[1]Regional Vascular and Endovascular Unit, Manchester Royal Infirmary, Central Manchester University Hospitals NHS Foundation Trust, Manchester Academic Health Science Centre, Oxford Road, Manchester, UK, M13 9WL.
[2] Institute of Cardiovascular Science, Manchester Academic Health Science Centre, University of Manchester, Core Technology Facility, 46 Grafton Street, Manchester, UK, M13 9NT.
[3]Translational Science, Healthcare Science Research Institute, Faculty of Science and Engineering, Manchester Metropolitan University, John Dalton Building, Chester Street, Manchester, UK, M1 5GD.
+these authors contributed equally to this work
* corresponding author; Y.Alexander@mmu.ac.uk, Tel: +44161247542

SUPPLEMENTARY MATERIAL

SUPPLEMENTARY TABLES

	Asymptomatic (n=19)	*Symptomatic (n=51)*	p Value
Age (average years) (Range)	67(48-87)	70 (54-83)	0.05
Male	13 (68%)	26 (51%)	0.23
Diabetes	6 (32%)	10 (20%)	0.28
Hypertension	16 (84%)	46 (90%)	0.50
Hypercholesterolemia	15 (79%)	43 (84%)	0.56
Coronary artery disease[a]	4 (21%)	23 (45%)	0.07
Renal Disease[b]	3 (16%)	8 (16%)	0.99
Anti-platelet agent	17 (89%)	49 (96%)	0.30
Anti-hypertensive	16 (84%)	46 (90%)	0.48
Statin	19 (100%)	48 (94%)	0.28
Smoking	13 (68%)	22 (43%)	0.06

Supplementary Table S1. Patient Demographics.
[a]Coronary disease defined as having a history of any of the following: arrhythmia, myocardial infarction, congestive heart failure, previous coronary revascularization (percutaneous or surgical) and angina. [b]Renal disease defined as patients with preoperative creatinine (pr-crt): $1.1 < \text{pr-crt} \leq 2.4$mg/dL or $2.5 \leq \text{pr-crt} \leq 5.9$mg/dL.

Duplex categories			
% ICA stenosis	50%	50-70%	70-99%
Asymptomatic	0	15	4
Symptomatic	7	26	18

Supplementary Table S2. Duplex ultrasonography categories. The percentage internal carotid artery (ICA) stenosis in asymptomatic and symptomatic patients was determined by duplex ultrasonography.

SUPPLEMENTARY FIGURES

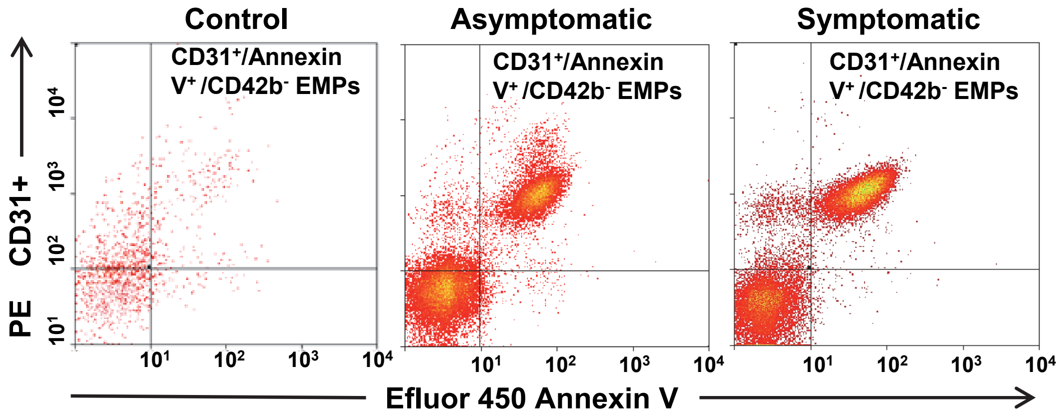

Supplementary Figure S1. Enumeration of EMPs from patients with carotid artery disease. Representative flow cytometry plots of each group. EMPS were defined as $CD31^+$/AnnexinV$^+$/$CD42^-$ and counted.

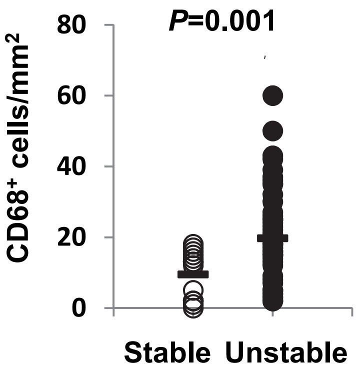

Supplementary Figure S2. Number of CD68-positive cells per mm^2 of stable and unstable plaques. Data were analysed using Mann-Whitney U statistical test. Bars represent the median.

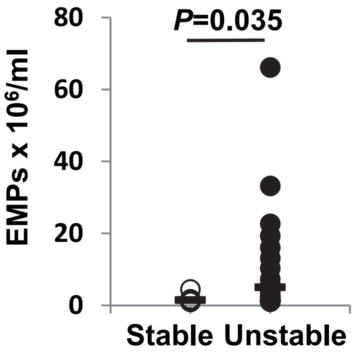

Supplementary Figure S3. EMPs were significantly elevated in patients with unstable plaques compared to those with stable plaques, when both symptomatic and asymptomatic groups were combined. Data were analysed using Mann-Whitney U statistical test. Bars represent the median.
